# Wild jujube-based fluorescent carbon dots for highly sensitive determination of oxalic acid†

**DOI:** 10.1039/d2ra03780g

**Published:** 2022-10-06

**Authors:** Huimin Shi, Xue Li, Yingying Li, Suling Feng

**Affiliations:** School of Chemistry and Chemical Engineering, Henan Normal University Xin Xiang 453007 China slfeng@htu.cn +86-373-3329250 +86-373-3326335; Department of Basic Medical Science, Zhengzhou Shuqing Medical College Zhengzhou 450064 China

## Abstract

Fluorescent carbon dots (CDs) were synthesized by a one-step hydrothermal treatment of wild jujube and dl-tryptophan. The structure and properties of the CDs were confirmed by transmission electron microscopy, X-ray photoelectron spectroscopy, ultraviolet visible absorption spectroscopy, fluorescence spectroscopy and so on. The as-prepared CDs exhibit excellent excitation-independent but pH-dependent (4.0–12.0) fluorescent features and emit blue strong fluorescence under 365 nm light. Hg^2+^ can decrease the fluorescence intensity of the CDs through static quenching, while the addition of oxalic acid (OA) recovers it owing to the coordination binding between oxalic acid and Hg^2+^. Based on this, the as-prepared CDs were used as a new “off–on” fluorescent probe for highly sensitive determination of oxalic acid with a wide linear detection range of 0.1–20 mg L^−1^ and a low detection limit of 0.057 mg L^−1^. Moreover, the fluorescent probe was successfully applied to detect oxalic acid in tomato and cherry tomato samples with satisfactory results.

## Abstract 2

Carbon dots synthesized from wild jujube and dl-tryptophan can serve as sensitive off–on fluorescent sensors for the detection of oxalic acid.

## Graphical abstract

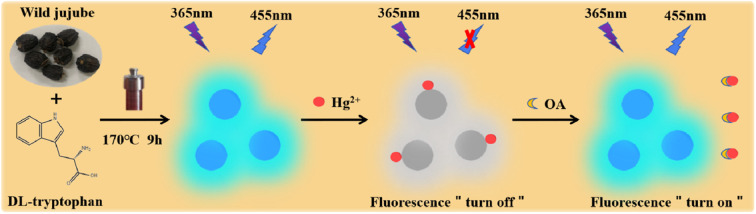

## 1. Introduction

Carbon dots (CDs) are an emerging type of zero-dimensional carbon nanomaterial with a size less than 10 nm. They have a lot of fascinating physical and chemical properties, including excellent photoluminescence, good biocompatibility, low toxicity, and chemical stability, among others.^1^ These superior properties endow CDs with wide use in the fields of fluorescent sensing,^2,3^ biological imaging,^4,5^ chemo-photothermal synergistic therapy,^6^ optoelectronic devices,^7^ photocatalysis,^8^ drug delivery,^9^ energy harvesting^10,11^ and others.

At present, there are many methods for synthesizing carbon dots, including arc discharge, laser ablation, electrochemical oxidation, ultrasonication, microwave pyrolysis, hydrothermal synthesis, *etc.*^12^ Out of these methods, the hydrothermal method, as the water-based synthesis pathway, has several advantages such as simplicity, low cost, environmental friendliness and so forth,^13^ and has received much attention from lots of researchers in recent years. Based on the hydrothermal method, CDs can be prepared from many carbon-containing materials, ranging from simple organic molecules,^14–16^ biomass^17–19^ to micro-organism,^20–22^ biomass waste^23–25^ and so on. Despite all this, the exploitation of high-quality carbon dots using low-cost, facile and eco-friendly precursors still faces great challenges. As a natural fruit with high nutritional and medicinal value, wild jujube contains polysaccharides, alkaloids, saponins, flavonoids, triterpenoids *et al.* Hence it is a good source of naturally occurring carbon.

Oxalic acid (OA) is the simplest organic dicarboxylic acid, namely ethanedioic acid, which is widely present in plants, animals and microorganisms.^26^ It can easily interact with calcium, magnesium, iron and so on to form less soluble oxalates. According to the report, 85% of urolithiasis mainly contain calcium oxalate.^27^ Too much ingestion of oxalic acid can lead to not only some diseases of the urinary system, but also hypocalcaemia, disturbing the activity of the heart and the neural system.^28^ Therefore, the detection of oxalic acid in food is particularly significant.

Up to now, many analytical methods have been developed for the detection of oxalic acid, such as the potassium permanganate titration method, colorimetric detection,^29^ spectrophotometry,^30^ chromatography,^31,32^ electrochemical detection,^33–35^ chemiluminescence,^36^ and so on. However, each method has usually suffered from diverse drawbacks, such as time-consuming, expensive equipment, insufficient selectivity, low sensitivity, and no real samples *etc.* Compared with these analysis technologies, fluorescence methods have attracted tremendous interest due to advantages of rapid response, high sensitivity, simplicity, and ease of operation *et al.* Some fluorescent methods have been established for the determination of oxalic acid, but most of them are based on the different metal complex, such as zinc-containing [DAQZ@2Zn^2+^] complex,^37^ dinuclear copper complex,^38^ and so on. Although CDs as fluorescent probes have been reported for the determination of oxalate,^39,40^ it is pity that the methods not only have inferior sensitivity, but also have not been used in the analysis of oxalic acid in real samples. So, it is still demanded to develop new fluorescence detection methods for the analysis of oxalic acid.

**Scheme 1:**
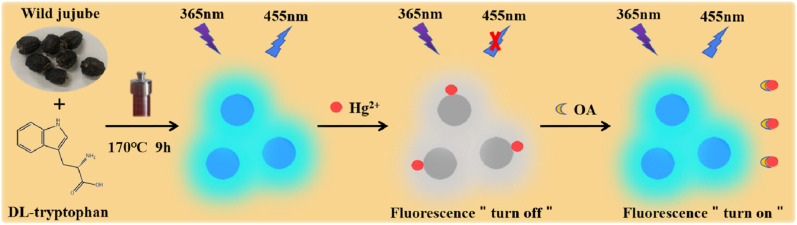
Schematic illustration of the synthesis of CDs and the detection mechanism of oxalic acid.

In the present work, the water-soluble fluorescent CDs were successfully prepared using wild jujube as a natural carbon source and dl-tryptophan as a nontoxic passivator through hydrothermal method. The synthetic method is facile, economic and eco-friendly. The fluorescence quantum yield of CDs is measured to be 16.87% and is superior to that of CDs from some other natural materials based on hydrothermal method.^14,22,41,42^ The fluorescence of CDs is decreased by Hg^2+^ through static quenching, while it is restored reversibly with the addition of oxalic acid by virtue of the coordination binding between Hg^2+^ and oxalic acid. Based on these, an effective off–on fluorescent sensor for the rapid detection of oxalic acid is established. The linear detection range for oxalic acid is 0.1–20 mg L^−1^ and the limit of detection is as low as 0.057 mg L^−1^. The present method exhibits higher sensitivity than some reported determination methods for oxalic acid,^29,30,33–35,39,40^ and has been successfully applied to detect oxalic acid in tomato and cherry tomato with good precision and accuracy. The schematic illustration of the synthesis of CDs and the detection mechanism of oxalic acid is demonstrated in Scheme 1.

## 2. Experimental

### 2.1. Reagents and chemicals

Wild jujube was picked from the mountain in Weihui, Xinxiang. dl-tryptophan was obtained from Sinopharm Chemical Reagent Co. Ltd (Beijing, China). Oxalic acid and mercuric chloride were acquired from Shanghai Fortunei Biotechnology Co. Ltd (China). A borax–boric acid–sodium chloride buffer solution was used to maintain the pH of solution at 6.4. All chemicals were analytical grade and used directly without further purification. Distilled water was used throughout the experiment.

### 2.2. Apparatus and characterization

All fluorescence measurements were acquired with FP-6500 fluorescence spectrophotometer (Hitachi, Tokyo, Japan). The fluorescence quantum yield (QY) was measured by FLS-980 fluorescent spectrophotometer (Edinburgh Instrument, UK). The UV-vis absorption spectra were acquired by TU-1810 UV spectrophotometer (Beijing Purkinje General Instrument Co. Ltd, China). X-ray diffraction (XRD) measurement was performed on a Bruker D8 ADVANCE X-ray diffractometer (Bruker AXS, German) with CuKα (1.54056 Å) as the incident radiation. X-ray photoelectron spectroscopy (XPS) were carried out using Thermo ESCALAB 250 (Thermo Electron Co., USA). Transmission electron microscope (TEM) images were observed by JEOLJEM-2010 transmission electron microscopy (JEOL, Japan). All pH adjustments were made using pHS-3C digital acidity meter (Shanghai INESA Scientific Instrument Co. Ltd, China). The CDs were centrifuged by TGL-16G centrifuge (Shanghai Anting Scientific Instrument Factory, China).

### 2.3. Synthesis of fluorescent CDs

The fluorescent CDs were prepared according to the following procedure:^43^ 1.0 g wild jujube and 0.5 g dl-tryptophan were added to 20 mL distilled water. The mixture was stirred well and then transferred to a 50 mL Teflon-lined autoclave and heated for 9 h at 170 °C. After being cooled to room temperature naturally, the resulting substances were centrifuged to eliminate large particles at 10 000 rpm for 10 min and then filtrated by 0.1 μm filter membrane. The obtained brown solution containing CDs were dried in vacuum drying box at 70 °C. Then the acquired solid CDs were dissolved in proper amount of water to form 0.74 g L^−1^ homogeneous solution and preserved at 4 °C for further use.

### 2.4. Detection of oxalic acid

In a 10 mL volumetric flask, 0.6 mL of 0.74 g L^−1^ CDs, 1.7 mL of pH 6.4 Na_2_B_4_O_7_–H_3_BO_3_–NaCl buffer solution, 1.0 mL of 1.0 mM HgCl_2_ and appropriate amount of oxalic acid working solution or sample solution were added to in turn. The mixtures were diluted to the mark with distilled water and then blended adequately. Subsequently, the fluorescence emission spectra of the system were recorded at the excitation wavelength of 365 nm with the excitation and emission slit widths of 5 nm. The fluorescent intensity was recorded under the maximum emission wavelength of 455 nm. The enhanced fluorescence intensity was calculated with the formula Δ*F* = *F* − *F*_0_, where *F* and *F*_0_ were the intensities of the system in the presence and absence of oxalic acid, respectively.

## 3. Results and discussion

### 3.1. Analysis of influencing factors on the preparation of CDs

Based on the literature,^43^ with 1.0 g wild jujube power and 20 mL distilled water, the fluorescence intensity of CDs is the highest when the reaction time is 9 h, reaction temperature is 170 °C and the amount of dl-tryptophan is 0.5 g. Too long reaction time or too high temperature as well as overmuch dl-tryptophan make the fluorescence of CDs decrease (Fig. S1†). The reason for the former two may be that excessive carbonization results in the decrease of fluorescence properties of CDs, while the latter is probably because that –NH_2_ of overmuch dl-tryptophan interacts with –COOH on the surface of CDs, which makes the CDs aggregate, leading to the decrease of fluorescence intensity.^44^

### 3.2. Characterization of CDs

**Fig. 1:**
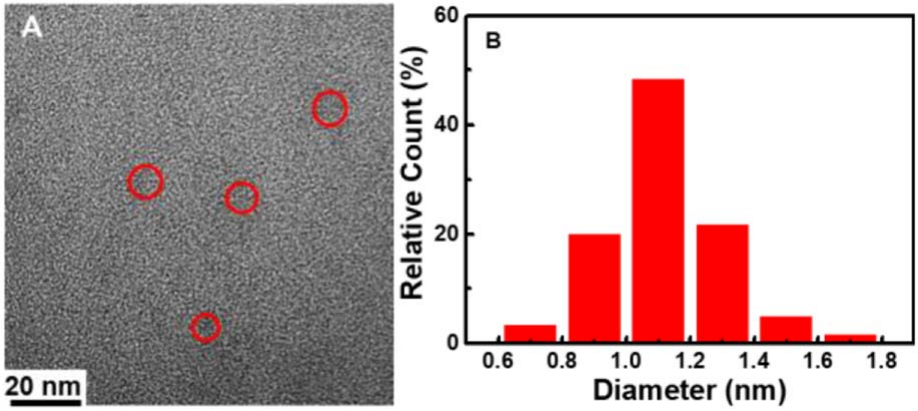
(A) TEM image of CDs. (B) Particle size distribution histogram.

**Fig. 2:**
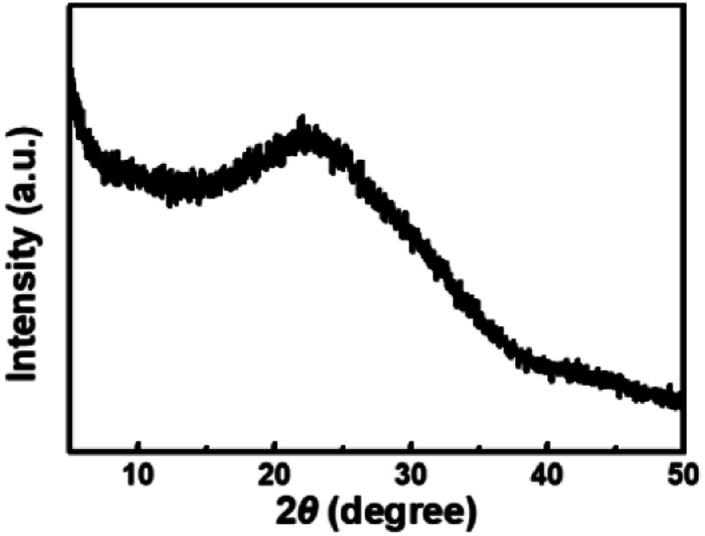
XRD pattern of CDs.

The size and morphology of CDs were characterized using TEM. It can be seen that the CDs are homogeneous and disperse well in a spherical shape (Fig. 1A). They have a relatively narrow particle size distribution ranging from 0.6 to 1.7 nm with an average diameter of 1.1 nm (Fig. 1B), which is smaller than the previously reported CDs.^14,41,42^ The XRD spectra (Fig. 2) was recorded in order to understand the crystal structure of CDs. It can be seen that there is a broad peak centered at 22.5°, which indicated an amorphous nature.^45^

**Fig. 3:**
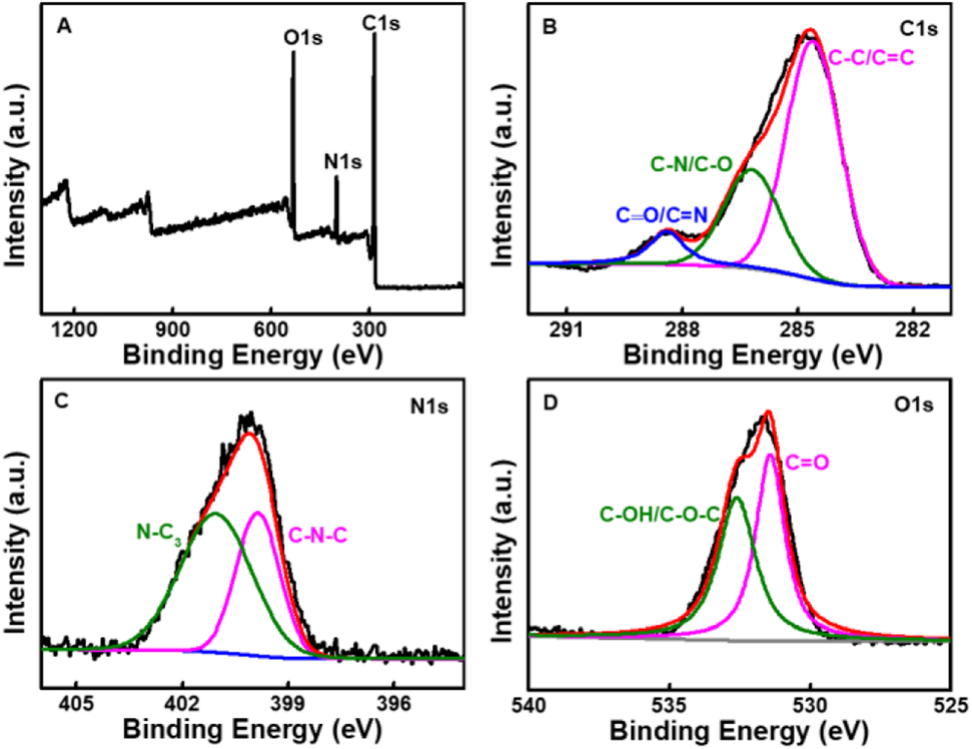
(A) Survey XPS spectrum of the CDs. High resolution XPS spectra of the C 1s (B), N 1s (C), O 1s (D) peaks of the CDs.

XPS was used to confirm the elemental composition and surface functional groups of the CDs. As shown in Fig. 3, the XPS spectrum presents three peaks at 284.8, 400.2, and 531.8 eV, which are ascribed to C, N, and O with the atomic contents of 69.7%, 11.2%, and 19.1%, respectively. The high-resolution spectrum of C 1s can be deconvoluted into three peaks at 284.6, 286.2, and 288.4 eV, corresponding to C–C/C=C, C–N/C–O, and C=O/C=N,^46^ respectively. The spectrum of N 1s exhibits two peaks, which are ascribed to C–N–C (399.3 eV) and N–(C)_3_ (400.7 eV) groups.^47^ The O 1s spectrum is separated into two peaks at 531.4 and 532.6 eV, which are assigned to C=O and C–OH/C–O–C^46^ chemical groups, respectively. These results show that there are characteristic groups like hydroxyl, carboxyl *et al.* on the surface of CDs, which can improve the hydrophilicity and stability of CDs.

### 3.3. Optical properties of CDs

For the purpose of studying the optical properties of the as-synthesized CDs, the UV-vis absorption, fluorescence excitation and emission spectra of CDs aqueous solution were scanned respectively.

**Fig. 4:**
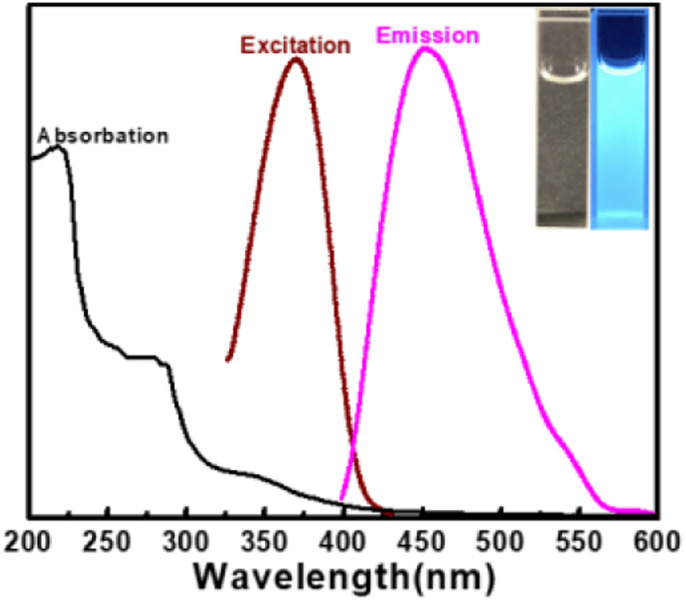
UV-vis absorption spectra and fluorescence spectra of CDs. Inset: photograph of CDs under daylight (left) and UV light (right).

As presented in Fig. 4, CDs have one peak at 225 nm and two shoulder peaks at 280 nm and 350 nm, with a tail spreading into the visible range. These peaks correspond to the π–π* transition of aromatic sp^2^ domains,^48^ the π–π* transition of the C=C bond^49^ and the n–π* transition of C=O^45^ on the surface of CDs, respectively. The shoulder peak at 350 nm should be put down to the trapping of excited state energy of the CDs surface states, leading to strong fluorescence emission.^44,50^ The aqueous solution of CDs exhibits obvious blue color under UV light of 365 nm (inset in Fig. 4). The optimum emission wavelength is at 455 nm with the optimum excitation wavelength 365 nm, which is very close to the absorption shoulder peak of 350 nm, suggesting that the fluorescence emission may be resulted from the band gap transition.^51^ The fluorescence lifetime of CDs is short to 8.11 ns,^43^ revealing the radiative recombination nature of excitations.^52^

The detailed fluorescence emission performance was evaluated under the different excitation wavelengths from 325–415 nm. As seen in Fig. S2A,† when the excitation wavelength changes, the fluorescence intensity of CDs first increases (excitation at 325–365 nm) and then decreases (excitation at 365–415 nm) and reaches the highest value at the excitation wavelength of 365 nm, suggesting that more particles are excited at 365 nm.^41^ However, the emission peak shows no evident shift and remains at 455 nm, showing that the CDs exhibit excitation-independent emission behavior, which can avoid auto fluorescence during their applications and also indicates that the prepared CDs have uniform surface state^46,53^ and size distribution.^54^ Moreover, the influence of emission wavelengths on fluorescence excitation intensities was also studied. Similar to the emission spectra of CDs, the fluorescence excitation intensity of CDs increases at first (emission at 420–450 nm) and then decreases (emission at 450–520 nm), but the excitation peak of CDs always keeps at 365 nm (Fig. S2B†). It is a sign of the emission-independent excitation behavior of CDs. Furthermore, at the maximum excitation wavelength of 365 nm, the absolute fluorescence quantum yield of CDs is measured to be 16.87%, which is better than that of other CDs originated from biomass materials.^14,22,41,42^

### 3.4. Stability of CDs

In order to investigate the stability of CDs, the effect of pH, ionic strength and illumination with a UV lamp on the fluorescence intensities of the as-prepared CDs were studied, respectively. Fig. S3A† shows that the fluorescence intensity of CDs is stable when pH increases from 2 to 4, while it significantly decreases according to the equation of *F* = −114.2 pH + 1330.8 (*R*^2^ = 0.962,^43^ Fig. S3B†) when pH is in the range of 4–12. This phenomenon is possibly attributed to the change of the surface charge due to the protonation–deprotonation of surface groups on CDs.^53^ The pH-dependent property makes it possible for CDs to act as a pH sensor. In addition, the fluorescence intensity of CDs exhibits obvious change under neither different NaCl concentrations (0–5.0 M) (Fig. S3C†) nor continuous irradiation at 365 nm with a UV lamp for 120 min (Fig. S3D†). The stability of CDs in high ionic strength solutions shows its application potential in high salt environments. All results mentioned above show that the as-prepared CDs exhibit good stability and are suitable for practical applications.

### 3.5. Effects of metal ions on the fluorescence of CDs

**Fig. 5:**
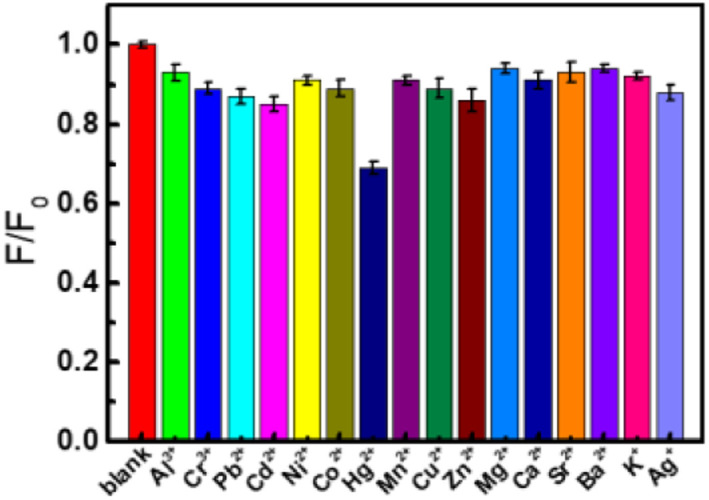
Histogram of the fluorescence intensity of the CDs in the absence and presence of different metal ions (CDs: 0.044 g L^−1^, M^*n*+^: 1.0 mM).

To evaluate the potential applications of CDs, the fluorescence intensities of CDs were analyzed with the existence of a series of 1.0 mM metal ions. As shown in Fig. 5, the examined metal ions (Al^3+^, Cr^3+^, Pb^2+^, Cd^2+^, Ni^2+^, Co^2+^, Mn^2+^, Cu^2+^, Zn^2+^, Mg^2+^, Ca^2+^, Sr^2+^, Ba^2+^, K^+^, Ag^+^) have a very slight quenching effect on the fluorescence of CDs (<15%). In comparation with the influence of these metal ions, Hg^2+^ shows relatively strong quenching to CDs (30%). Maybe it is because that Hg^2+^ have a stronger binding affinity and faster chelating kinetics with CDs than other metal ions.^55,56^ Based on the fluorescence quenching of Hg^2+^ to the as-prepared CDs, a highly sensitive method for Hg^2+^ was realized.^43^

### 3.6. Optimization of conditions for the detection of oxalic acid

In order to obtain the highest detection sensitivity of oxalic acid, the experimental conditions including the type and amount of buffer solution, pH, the concentration of Hg^2+^ and CDs as well as the addition sequence of reagents were optimized. Among six buffer solutions (Na_2_B_4_O_7_–H_3_BO_3_–NaCl, NaOH–KH_2_PO_4_, KH_2_PO_4_–Na_2_HPO_4_, KH_2_PO_4_–borax, Na_2_HPO_4_–citric acid, Britton–Robinson), Na_2_B_4_O_7_–H_3_BO_3_–NaCl can give the highest sensitivity of the system. Then, the effects of pH and amount of Na_2_B_4_O_7_–H_3_BO_3_–NaCl on the detection sensitivity were tested. It can be seen from Fig. S4A and B† that when 1.7 mL of Na_2_B_4_O_7_–H_3_BO_3_–NaCl buffer solution with pH 6.4 is used, the enhanced fluorescence intensity (Δ*F*) reaches the maximum. Furthermore, the effect of Hg^2+^ and CDs concentration on fluorescence intensity of the system were studied respectively. As shown in Fig. S4C and D,† 0.1 mM of Hg^2+^ and 0.044 g L^−1^ of CDs give the highest Δ*F*. As for the addition sequence of reagents, the experiment result shows that it has little effect on the detection sensitivity. To sum up, 1.7 mL of pH 6.4 Na_2_B_4_O_7_–H_3_BO_3_–NaCl buffer solution, 0.1 mM of Hg^2+^ and 0.044 g L^−1^ of CDs were used as the optimum conditions for the detection of oxalic acid according to the sequence of CDs + Na_2_B_4_O_7_–H_3_BO_3_–NaCl + Hg^2+^ + OA.

### 3.7. Calibration and sensitivity

**Fig. 6:**
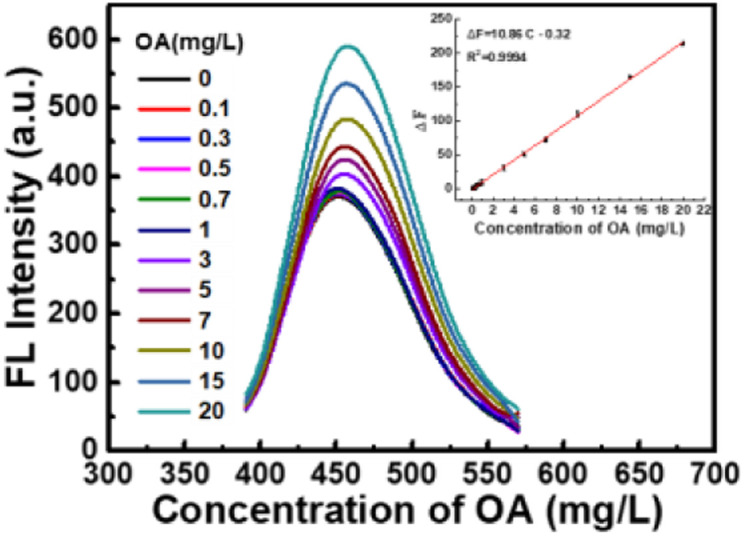
Fluorescence response of CDs–Hg^2+^ in the presence of increasing concentration of OA (from bottom to top: 0, 0.1, 0.3, 0.5, 0.7, 1, 3, 5, 7, 10, 15, 20 mg L^−1^). Inset: the linear relationship of Δ*F versus* the concentration of OA. (CDs: 0.044 g L^−1^, Hg^2+^: 0.1 mM, Na_2_B_4_O_7_–H_3_BO_3_–NaCl: pH 6.4, 1.7 mL).

Under the above-mentioned optimum conditions, the fluorescence spectra of CDs–Hg^2+^ system in the presence of various concentrations of oxalic acid were studied. As shown in Fig. 6, the fluorescence intensity of the system enhances gradually with the concentration of oxalic acid increase. There is a good linear relationship between the enhanced fluorescence intensity (Δ*F*) and the concentration of oxalic acid in the range of 0.1 mg L^−1^ to 20 mg L^−1^. The corresponding linear regression equation is Δ*F* = 10.86*c* − 0.32 (*r* = 0.9994, *n* = 11). The detection limit calculated according to the 3*σ* is 0.057 mg L^−1^. The relative standard deviation (RSD) of 11 parallel determinations of 10 mg L^−1^ oxalic acid is 0.13%, indicating the excellent repeatability of the proposed method for oxalic acid detection. Compared with some previous reports as shown in Table 1, the proposed method has wider linear detection range and lower detection limit, which provide the foundation of application in real samples.

**Table 1:** Comparison of the proposed method for the determination of oxalic acid with others

Detection method	Electrode/probe	Linear range (mM)	LOD (μM)	Reference
Colorimetric detection	TMB-MnO_2_^a^	0.0078–0.25	0.91	29
Spectrophotometry	Zr(iv)-(DBS-ASA)^b^	0.009–0.5	9.1	30
Electrochemistry	Graphite/Ag/AgCl nanocomposite	0.01–0.75	3.7	33
Electrochemistry	NH_2_-GQD^c^	0.5–2.0, 2.0–55	50	34
Electrochemistry	Gr-Ag NRs^d^	3–30	40	35
Fluorescence	Carbon dots	0.01–0.07	1.0	39
Fluorescence	Carbon dots	0–0.045	3.5	40
Fluorescence	Carbon dots	0.0011–0.22	0.63	This work

a 3,3′,5,5′-tetramethylbenzidine–manganese dioxide.

b Zirconium(iv)-(DBS-arsenazo).

c Amino-functionalized graphene quantum dots.

d graphene (Gr) functionalized with silver nanorods (Ag NRs).

### 3.8. Interference of foreign coexisted substances

**Fig. 7:**
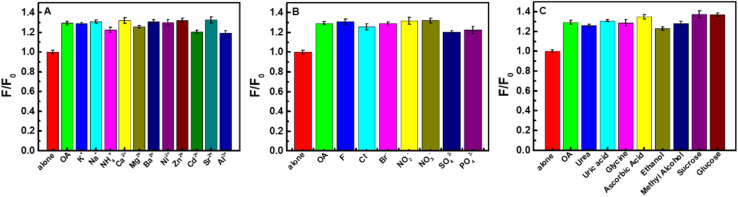
Influence of potential interfering species on the determination of OA. (A) cations, (B) anions, (C) organics (CDs: 0.044 g L^−1^, Hg^2+^: 0.1 mM, OA: 1.0 mg L^−1^, interfering species: 100 mg L^−1^, Na_2_B_4_O_7_–H_3_BO_3_–NaCl: pH 6.4, 1.7 mL).

In order to investigate the anti-interference of the CDs–Hg^2+^ sensing system, the effects of some potential foreign coexisted substances including cations (K^+^, Na^+^, NH_4_^+^, Ca^2+^, Mg^2+^, Ba^2+^, Ni^2+^, Zn^2+^, Cd^2+^, Sr^2+^, Al^3+^), anions (F^−^, Cl^−^, Br^−^, NO_2_^−^, NO_3_^−^, SO_4_^2−^, PO_4_^3−^) and organics (urea, uric acid, glycine, ascorbic acid, ethanol, methyl alcohol, sucrose, glucose) on the detection of 1.0 mg L^−1^ oxalic acid were researched. It can be observed from Fig. 7 that even with 100-fold amount, the influence of above-mentioned coexisting substances shows negligible effect. Consequently, the CDs–Hg^2+^ system can be expected to be used for the detection of oxalic acid in practical samples.

### 3.9. Possible mechanism

**Fig. 8:**
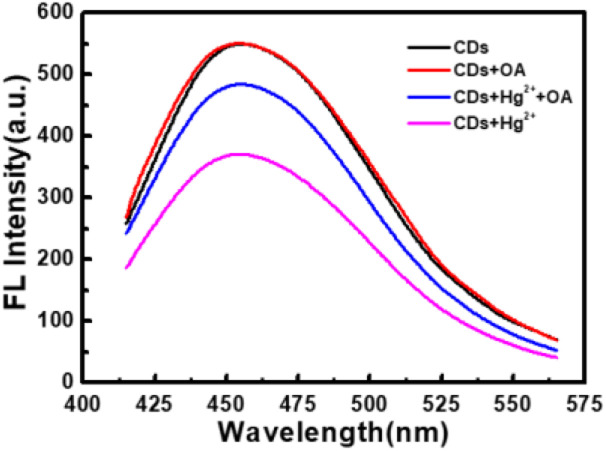
Fluorescence spectra of CDs (black curve), CDs + OA (red curve), CDs + Hg^2+^ + OA (blue curve) and CDs + Hg^2+^ (magenta curve). (CDs: 0.044 g L^−1^, Hg^2+^: 0.1 mM, OA: 10 mg L^−1^, Na_2_B_4_O_7_–H_3_BO_3_–NaCl: pH 6.4, 1.7 mL).

**Fig. 9:**
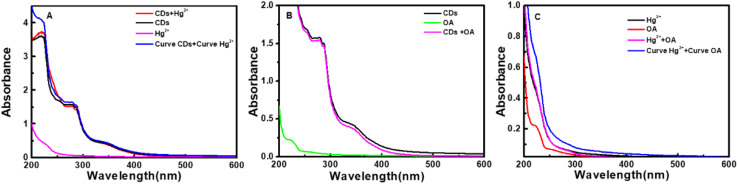
(A) Absorption spectra of CDs (black curve), Hg^2+^ (magenta curve), CDs + Hg^2+^ (experimental one: red curve, theoretical one: blue curve). (B) Absorption spectra of CDs (black curve), OA (green line), CDs + OA (magenta curve). (C) Absorption spectra of Hg^2+^ (black curve), OA (red curve), Hg^2+^ + OA (experimental one: magenta curve, theoretical one: blue curve). (CDs: 0.044 g L^−1^, OA: 10 mg L^−1^, Hg^2+^: 0.1 mM, Na_2_B_4_O_7_–H_3_BO_3_–NaCl: pH 6.4, 1.7 mL).

**Fig. 10:**
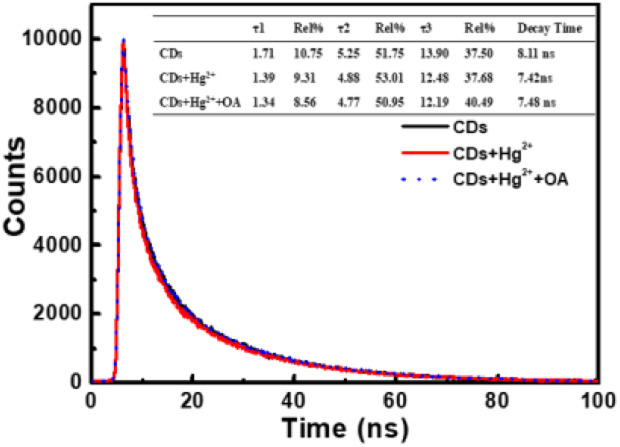
Time-resolved fluorescence decays of CDs, CDs + Hg^2+^ and CDs + Hg^2+^ + OA.

It can be seen from Fig. 8 that CDs exhibit a strong fluorescence peak at 455 nm with the excitation wavelength at 365 nm. While the introducing of Hg^2+^ leads to an evident decrease of the fluorescence intensity of CDs, elucidating that Hg^2+^ can effectively quench the fluorescence of CDs. In order to investigate the quenching fluorescence mechanism, the UV-vis absorption spectrum of CDs was scanned in the presence of Hg^2+^ (Fig. 9A). When Hg^2+^ coexists with CDs, the shape of absorption spectrum of CDs around 230–290 nm is changed, and the experimental spectrum of CDs–Hg^2+^ is obviously different from the theoretical one. These phenomena prove the interaction between CDs and Hg^2+^, and the fluorescence quenching of CDs induced by Hg^2+^ may be the static quenching. This is a new discovery and is different from the previously reported conclusion of dynamic quenching,^43^ where no changes about the absorption spectra of CDs caused by Hg^2+^ were found. To further ascertain the quenching mechanism, the fluorescence lifetime was measured (Fig. 10). The fluorescence decay of CDs is fitted with a three exponential decay function. The average lifetime of CDs is 8.11 ns and it only decays to 7.42 ns with the addition of Hg^2+^.^43^ The decrease is too little. Combined with the above discussion results of UV spectra, the so little decrease of fluorescence lifetime indicates to a static quenching.^46^ Possibly, the reason is that the presence of functional groups (carboxy and hydroxyl *etc.*) on the surface of CDs can interact with Hg^2+^ to form nonfluorescent complexes, which causes the non-radiative electron transfer from CDs to Hg^2+^, leading to a quenching effect.

Furthermore, as shown in Fig. 9B, the addition of oxalic acid hardly changes the UV-vis absorption spectrum of CDs, indicating that there is no interaction between oxalic acid and CDs, which can be supported by the negligible change between the emission spectra of CDs and that of CDs–OA (Fig. 8). However, when oxalic acid and Hg^2+^ coexist, not only the experimental absorption curve of Hg^2+^–OA (Fig. 9C, magenta curve) is obviously different from the theoretical one (Fig. 9C, blue curve), but also the π–π* corresponding absorption bands of oxalic acid enhance, suggesting the interaction between OA and Hg^2+^.

For the CDs–Hg^2+^ system, oxalic acid makes the fluorescence intensity recovering significantly (Fig. 8). Maybe it is because that under the experimental condition of pH 6.4, oxalic acid (p*K*_a1_ = 1.2, p*K*_a2_ = 4.2) is mainly present in the form of C_2_O_4_^2−^ and the presence of oxygen atoms in C_2_O_4_^2−^ can coordinate with Hg^2+^,^38^ which may be relatively stronger than the interaction between CDs and Hg^2+^, resulting in the release of CDs and then the restoration of fluorescence.

### 3.10. Analytical application

The feasibility of CDs–Hg^2+^ system for detecting oxalic acid in real samples was validated by tomato and cherry tomato samples. In the light of the reference,^30^ 50 g tomato or cherry tomato sample was accurately weighed, then cut up and soaked it with 250 mL boiling distilled water for 30 min. After the mixture cooled off, it was filtered for 3 times. The filter liquor was transferred into the 250 mL volumetric flask and added distilled water to the scale mark. Then the solution was diluted 10 times with distilled water. Subsequently, an amount of the above-mentioned sample solution was taken to test the content of oxalic acid following the general procedure. The results are list in Table 2. The mean contents of oxalic acid in tomato 1, tomato 2 and cherry tomato 1 samples are found to be 1.316 mg g^−1^, 1.351 mg g^−1^ and 1.633 mg g^−1^, respectively. The recoveries of the developed method are in the range of 91.07–105.3% with the RSD of the results within 0.34%, illuminating the better accuracy and precision of the proposed method.

**Table 2:** Results for the determination of oxalic acid in real samples (*n* = 6)

Samples	Found (mg g^−1^)	Added (mg g^−1^)	Total found (mg g^−1^)	Recovery (%)	RSD (%)
Tomato 1	1.316	4.000	5.222	97.65	0.34
		1.000	2.369	105.3	0.29
Tomato 2	1.351	1.000	2.351	100.0	0.32
		0.2500	1.613	104.8	0.14
Cherry tomato 1	1.633	0.7500	2.316	91.07	0.22
		0.3333	1.971	101.4	0.16

## 4. Conclusion

We prepared water-soluble fluorescent CDs utilizing wild jujube and dl-tryptophan by hydrothermal method. The synthesized CDs emits blue fluorescence with high fluorescence quantum yield of 16.87% and exhibits monodispersed mostly spherical morphology and excellent stability. Hg^2+^ can reduce the fluorescence of CDs through static quenching, whereas in the presence of oxalic acid, the coordination between Hg^2+^ and C_2_O_4_^2−^ induces the fluorescence recovery of the quenched CDs. On the basis of this, the CDs–Hg^2+^ system was utilized as fluorescent probe for the sensitive determination of oxalic acid with a detection limit of 0.057 mg L^−1^. And the probe has been used to determine oxalic acid in tomato and cherry tomato samples with good accuracy and precision, which may extend the potential application of CDs in food monitoring fields.

## Conflicts of interest

There are no conflicts of interest to declare.

## Supplementary Material

RA-012-D2RA03780G-s001
