# Supplementary material for: Wild jujube-based fluorescent carbon dots for highly sensitive determination of oxalic acid
Source: RSC Adv. 2022 Oct 6;12(44):28545–52. doi: 10.1039/d2ra03780g (PMC9535697; doi:10.1039/d2ra03780g)
Supplement: RA-012-D2RA03780G-s001 [file RA-012-D2RA03780G-s001.pdf]

Electronic Supplementary Material (ESI) for RSC Advances.
This journal is © The Royal Society of Chemistry 2022

Supporting Information

Wild jujube-based fluorescent carbon dots for highly sensitive determination of oxalic acid

Huimin Shi,[a, b] Xue Li,[a] Yingying Li[a] and Suling Feng*[a]

[a] School of Chemistry and Chemical Engineering, Henan Normal University, Xin Xiang 453007, China

[b] Department of Basic Medical Science, Zhengzhou Shuqing Medical College, Zhengzhou 450064, China

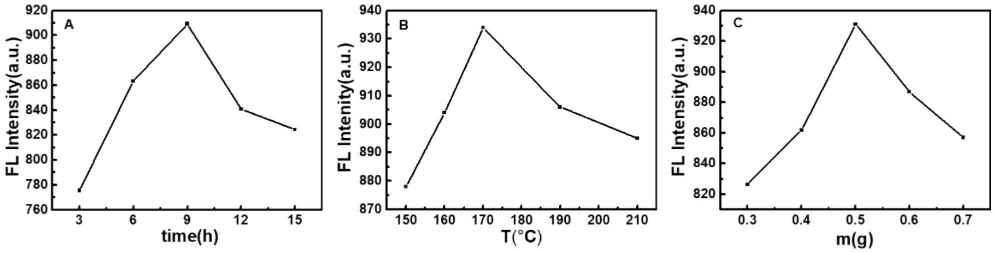

Fig. S1 (A) The effects of reaction time on the fluorescence intensity of CDs. (Wild jujube: 1.0 g, DL-tryptophan: 0.5 g, reaction temperature: 170 °C). (B) The effects of reaction temperature on the fluorescence intensity of CDs. (Wild jujube: 1.0 g, DL-tryptophan: 0.5 g, reaction time: 9 h). (C) The effects of the amount of DL-tryptophan on the fluorescence intensity of CDs. (Wild jujube: 1.0 g, reaction temperature: 170 °C, reaction time: 9 h).

* Corresponding author. Tel.: +86-373-3326335; fax: +86-373-3329250. *E-mail address*: slfeng@htu.cn

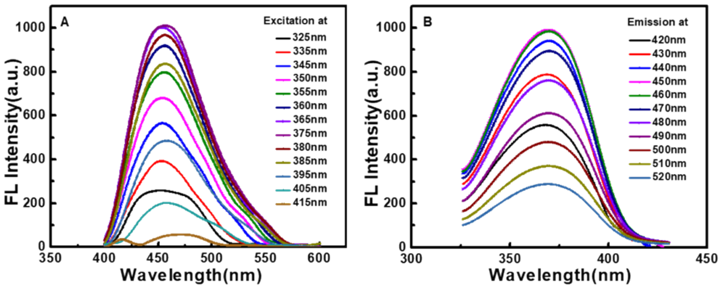

Fig. S2 (A) Fluorescence emission spectra of CDs with the excitation wavelength increasing from 325 to 415 nm. (B) Fluorescence excitation spectra of CDs with the emission wavelength increasing from 420 to 520 nm.

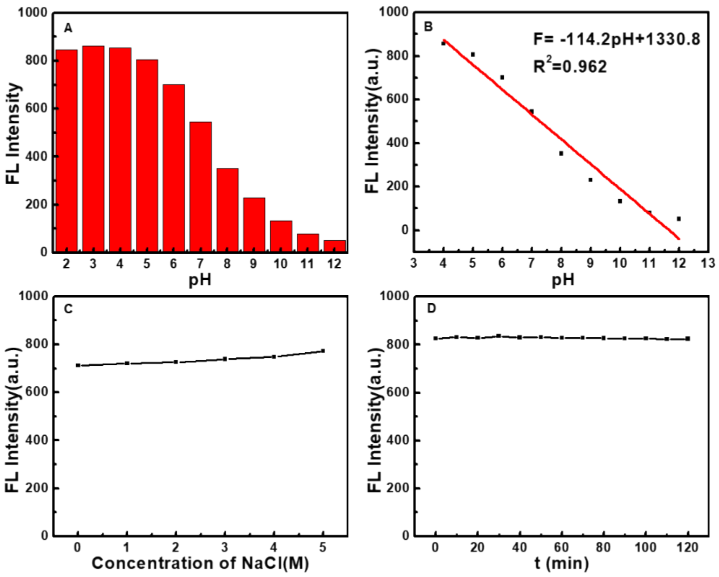

Fig. S3 (A) Fluorescence intensity of CDs in different pH. (B) The relationship between the fluorescence intensity of CDs and pH variation in the range of 4.0-12.0. (C) The effects of the concentration of NaCl on the fluorescence intensity of CDs. (D) The stability of CDs under UV light illumination.

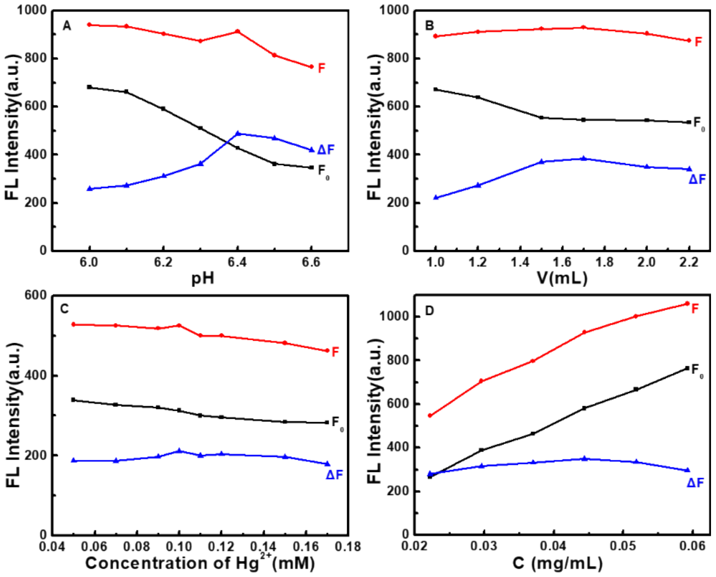

Fig. S4 (A) The effect of pH on the fluorescence intensity. (CDs: 0.044 g L^{-1}, Hg^{2+}: 0.1 mM, OA: 0.02 g L^{-1}, $Na_2B_4O_7$-H_3BO_3-NaCl: 1.7 mL) (B) The effect of the amount of $Na_2B_4O_7$-H_3BO_3-NaCl on the fluorescence intensity. (CDs: 0.044 g L^{-1}, Hg^{2+}: 0.1 mM, OA: 0.02 g L^{-1}, $Na_2B_4O_7$-H_3BO_3-NaCl: pH 6.4.) (C) The effect of the concentration of Hg^{2+} on the fluorescence intensity. (CDs: 0.044 g L^{-1}, OA: 0.02 g L^{-1}, $Na_2B_4O_7$-H_3BO_3-NaCl: pH 6.4, 1.7 mL) (D) The effect of the concentration of CDs on the fluorescence intensity. (Hg^{2+}: 0.1 mM, OA: 0.02 g L^{-1}, $Na_2B_4O_7$-H_3BO_3-NaCl: pH 6.4, 1.7 mL). (F: the fluorescence intensity of CDs-Hg^{2+}-OA, F_0: the fluorescence intensity of CDs-Hg^{2+}, $\Delta F = F - F_0$).
